# Anti-Cancer Drugs Elicit Re-Expression of UDP-Glucuronosyltransferases in Melanoma Cells

**DOI:** 10.1371/journal.pone.0047696

**Published:** 2012-10-22

**Authors:** Ryan W. Dellinger, Harry H. Matundan, Amelia S. Ahmed, Priscilla H. Duong, Frank L. Meyskens

**Affiliations:** 1 Chao Family Comprehensive Cancer Center, University of California Irvine, Irvine, California, United States of America; 2 Department of Medicine, University of California Irvine, Irvine, California, United States of America; 3 Department of Biological Chemistry, University of California Irvine, Irvine, California, United States of America; 4 Department of Public Health, University of California Irvine, Irvine, California, United States of America; The Moffitt Cancer Center & Research Institute, United States of America

## Abstract

The UDP-glucuronosyltransferase (UGT) family of enzymes plays a vital role in the detoxification of carcinogens as well as clearance of anti-cancer drugs. In humans, 19 UGT family members have been identified and are expressed in a tissue specific manner throughout the body. However, the UGTs have not been previously characterized in melanocytes or melanoma. In the present study, UGT2B7, UGT2B10, and UGT2B15 were identified as being normally expressed in human melanocytes. The same three UGT family members were also expressed in the primary melanoma cell line WM115. No UGT expression was detected in another primary melanoma cell line, WM3211, or in any metastatic melanoma cell line examined. These results suggest that UGT expression is lost during melanoma progression. Treatment of WM3211 or metastatic melanoma cell lines with anti-cancer agents (including vemurafenib) induced expression of UGT2B7, UGT2B10 and UGT2B15 demonstrating that melanoma cells retain the ability to re-express these same three UGTs. The corresponding increase in glucuronidation activity in melanoma cells following anti-cancer treatment was also observed. Furthermore, knockdown of UGT2B7 in WM115 cells sensitized these cells to treatment by adriamycin and epirubicin indicating that UGT2B7 is involved in resistance to these drugs. However, knockdown of UGT2B7 had no effect on temozolomide toxicity. Taken together, these results clearly demonstrate a role for UGTs in melanoma etiology. Since the UGTs are drug metabolism enzymes, we propose that re-expression of the UGTs constitutes a previously unsuspected mechanism for intratumoral drug resistance in melanoma.

## Introduction

The UGT family of enzymes catalyzes the glucuronidation of a wide range of xenobiotic and endogenous compounds. UGTs conjugate a glucuronic acid moiety to their substrates, altering the biological properties of the substrate and enhancing its excretion in urine or bile [1], [2]. In general, glucuronidation converts substrates into less bioactive, more water soluble products facilitating their removal from the body. In this manner, the UGTs are integrally involved in the detoxification of many carcinogens, the clearance of drugs and the metabolism of a variety of endogenous substrates such as bilirubin, steroid hormones and bioactive lipids [1], [2]. There are 19 functional human UGTs classified into three subfamilies based upon structural and amino acid sequence homology, UGT1A, UGT2A and UGT2B [2].

The UGTs are membrane bound enzymes largely localized to the endoplasmic reticulum [1], [3]. Substrate specificity varies greatly between family members, with broad overlap, and their substrate specificity can be altered by posttranslational modifications such as phosphorylation [4]. Although UGTs are primarily expressed in liver, they also play vital roles in other tissues in the body. For example, UGT2B15 and UGT2B17 are expressed in the prostate where they regulate local androgen levels through glucuronidation [5] and UGT1A10 and UGT2B7 are expressed in the breast where they regulate estrogens [6]. There is also ample evidence that the UGTs play important roles in the aerodigestive tract, gastrointestinal tract, lung, colon, bladder, kidneys and brain [7], [8], [9], [10], [11]. However, the role of the UGTs in skin, the largest organ in the body, has yet to be investigated.

Melanoma is one of the fastest growing tumor types in the United States and the number of cases worldwide has doubled in the past 30 years [12]. Melanoma, which arises from melanocytes, is an extremely aggressive tumor that invades the vascular and lymphatic systems to form tumors elsewhere in the body [12], [13], [14]. Melanoma is a particularly resilient cancer, accounting for only 4% of all skin cancers but responsible for 80% of skin cancer deaths [15]. Further, only 14% of patients with metastatic melanoma survive for 5 years [15].

Systemic therapy approaches have achieved minimal success against metastatic melanoma resulting in only a few FDA-approved treatments [16]. Interferon-α2b, interleukin-2 and temozolomide have all demonstrated limited efficacy with response rates generally under 15% in the short term with no clear effect on melanoma-related mortality [16]. However, the recent success of the specific BRAF mutant inhibitor vemurafenib in a Phase 1 clinical trail is very promising [17]. An estimated progression free survival of 7 months was reported among all patients harboring the BRAF V600E mutation [17], which is present in approximately 50% of all melanomas [18]. However, the excitement for vemurafenib as a single agent has been tempered somewhat since acquired resistance is already being observed [17]. Thus, understanding the mechanism(s) of resistance to chemotherapy that melanoma cells employ is paramount to combating this deadly disease.

The goal of the present study was to characterize the expression and function of UGTs in melanocytes and melanoma and to examine the potential role of UGTs in drug resistance. Evidence presented here reveals three UGT family members, UGT2B7, UGT2B10 and UGT2B15, as being normally expressed in human melanocytes isolated from neonatal foreskins. The same three UGTs were found to be expressed in the primary melanoma cell line WM115. Interestingly, no UGT expression was observed in another primary melanoma cell line, WM3211, or in any of the three metastatic melanoma cell lines examined, suggesting that UGT expression is lost during melanoma progression. However, treatment of melanoma cells with anti-cancer agents results in re-expression of these same three UGTs. This novel re-expression of UGTs in melanoma is investigated further herein.

## Materials and Methods

### Reagents and Cell Culture

Temozolomide, adriamycin, epirubicin and alamethicin were obtained from Sigma. Vemurafenib was purchased from Selleck (Houston, TX). Normal human melanocytes were isolated from de-identified newborn foreskin from circumcision surgery in accordance with a protocol approved by UC Irvine’s Internal Review Board. In accordance with this approved protocol there is no recruitment of subjects, only discarded circumcised tissue is collected. No identifying information is collected regarding this tissue that would otherwise be discarded. Melanocytes were isolated as previously described [19], [20] and cultured in MCDB153 media supplemented with 2% fetal bovine serum, 10 ng/ml of 12-O-tetradecanoylphobol-13-acetate and 0.15% bovine pituitary extract. The melanoma cell lines WM115, WM3211, Lu1205, SKmel28 and A375 were cultured as described previously [21], [22], [23]. Only WM3211 has WT B-Raf, all others harbor V600E mutations. All cell lines tested negative for mycoplasma.

### Total RNA Isolation, Reverse Transcription and PCR

Total RNA was isolated from cells using the Arum total RNA mini Kit (BioRad) according to companies provided protocol. RNA was quantitated using a NanoDrop 1000 (Thermo/Fisher) cDNA was then made from 1.0 µg of RNA using the iScript Reverse Transcriptase Kit (BioRad) according to standard protocols. PCR was then performed using a Quick load Taq (2X) master mix (New England Biolabs) and run on an Applied Biosystems GeneAmp System 2700 thermocycler using the following protocol: Step 1 = 94°C for 5 min; Step 2 (40 cycles) = 94°C for 30 Sec, 55°C for 30 Sec, 68°C for 1 min; Step 3 = 68°C for 10 min. Table S1 in supplementary data lists the primers used to amplify individual UGT family members.

### Real-Time PCR

To analyze UGT mRNA expression levels in melanoma cells real-time PCR was performed as previously described [3], [24]. Briefly, pre-designed TaqMan Gene Expression Assays [Applied Biosystems (ID’s Hs02383831_s1 for UGT2B4; Hs00426592_m1 for UGT2B7; Hs02556282_s1 for UGT2B10; Hs03008769_g1 for UGT2B15 and Hs99999905_m1 for GAPDH)] were used according to manufacturer’s protocol. Real-time PCR was performed using a total volume of 20 µl containing 50 ng of cDNA using GAPDH as the normalizing ‘housekeeping’ gene. Real-time PCR was performed on a CFX96 Real-Time PCR machine (BioRad). Reported mRNA expression values are the average of at least 3 independent experiments with standard deviation.

### shRNA

Ready-cloned shRNA targeted against UGT2B7 and control empty expression vectors were purchased Origene. Each shRNA vector was transfected into WM115 cells using the BioT transfection agent (Bioland Scientific) according to manufacturer protocols and stable expression was selected for by addition of puromycin (Invivogen).

### MTT Assay

To determine the effects of drugs on cell proliferation we performed 3-(4,5-dimethylthiazol-2-yl-2,5-diphenyltetrazolium bromide (MTT) assays (Sigma Aldrich). Briefly, cells were plated overnight at 2000–4000 cells/well (depending on cell doubling times) in 96 well plates, and treated with either adriamycin, epirubicin, or temozolomide at serial dilutions of the drug. After the third day post-treatment, MTT diluted in dye-free media is added to each well and incubated for 3 hrs. Once the MTT is metabolized by actively proliferating cells into water insoluble formazen then, dimethylsulfoxide (Sigma Aldrich) is added and colorimetric changes are analyzed and quantified with a spectrophotometer at 590 nm. Results from MTTs were analyzed using Graphpad Prism where the dosage of the drug versus its effect on proliferation is graphed; a non-linear regression curve using the sigmoidal dose-response equation was fitted onto the graph and the half-maximal inhibitory concentration (IC_50_) is obtained. Reported IC_50_ values are the average of at least 3 independent experiments with standard deviation.

### UGT Activity Assay

UGT activity was examined using the UGT-Glo assay (Promega) according to manufacturer protocols. Cell homogenates were prepared by re-suspending pelleted cells in Tris-buffered saline (25 mM Tris base, 138 mM NaCl and 2.7 mM KCl; pH 7.4) and subjecting them to three rounds of freeze–thaw prior to homogenization using a glass dounce homogenizer. Cell homogenates (5–20 mg protein/ml) were stored at −70°C in 100 µl aliquots. Total cell homogenate protein concentrations were determined using the BCA assay from Pierce Biotechnology (Rockford, IL) after protein extraction using standard protocols. Human liver microsomes (Xenotech, Lenexa, KS) were used as a positive control. Homogenates were incubated with alamethicin for 10 minutes on ice. Each reaction consisted of 50 µg homogenate, UGT-Glo buffer, 50 µM UGT Multienzyme Substrate and water to a total of 30 µl. Then either 10 µl of water or 10 µl 16 mM UDPGA (final concentration 4 mM) was added. Each of these reactions was done in triplicate (total of 6 reactions for each homogenate). Reactions were then incubated at 37°C for 90 min. Only the reactions with the co-substrate UDPGA added can glucuronidate substrate. After 90 min, 40 µl of Luciferin Detection Reagent plus D-Cysteine is added to all wells and the luminescent signal is allowed to stabilize at room temperature for 20 min. Plate was then read on a luminometer (Turner Biosystems modulus microplate). As a negative control one set of six reactions was performed without any UGTs present. The UGT Multienzyme Substrate will generate luminescence, but the glucuronidated UGT multienzyme substrate will not. Thus, the average of the triplicate reactions with the co-substrate UDPGA is subtracted from the average of the triplicate reactions without UDPGA to determine UGT activity. Graphs are the composite of multiple independent experiments with standard deviation.

## Results

### UGT Expression in Human Melanocytes and Melanoma

**Figure 1:**
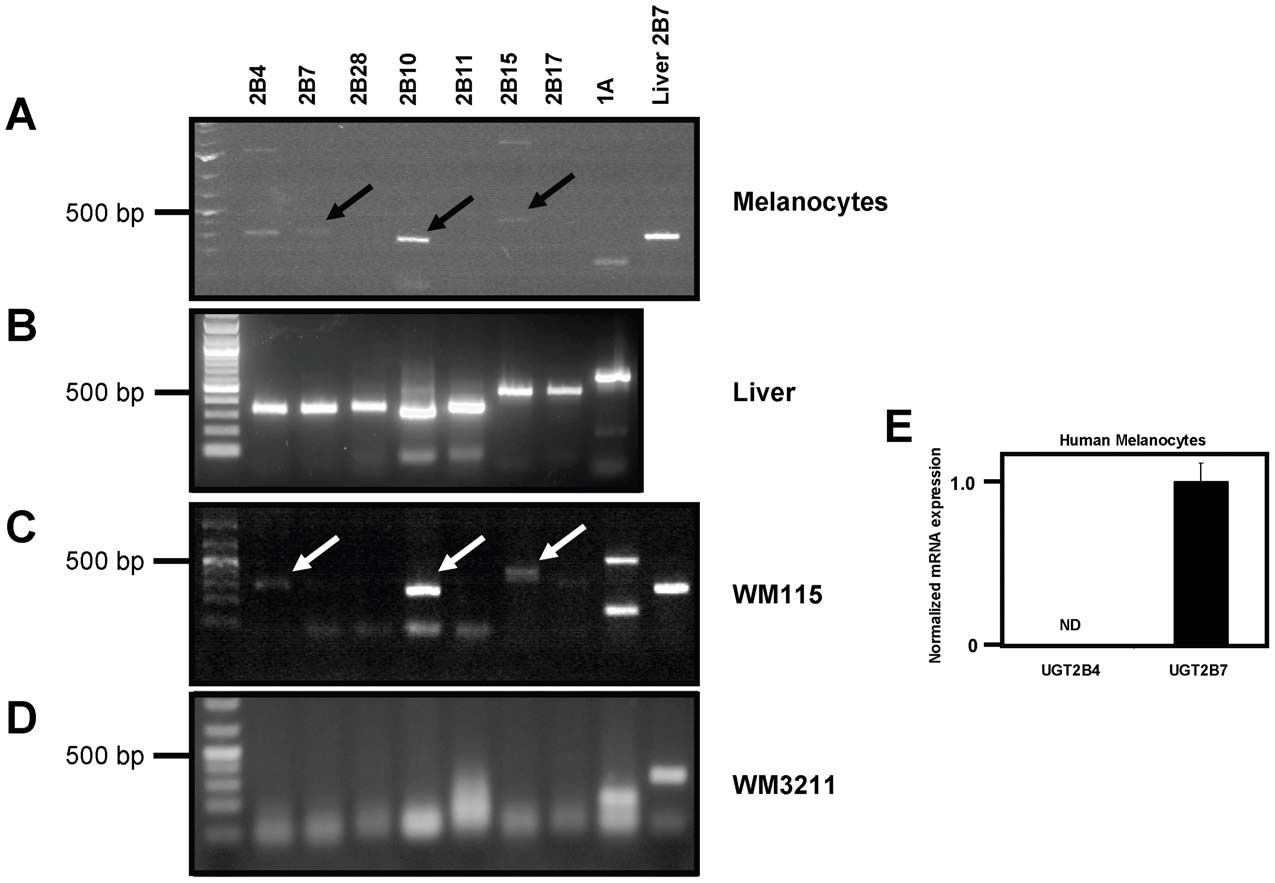
UGT mRNA expression in primary human melanocytes and melanoma. (A) RT-PCR analysis of total RNA from human melanocytes for UGT family members. Primer sets for the UGT2Bs were designed to be specific to each isoform while a single primer set directed against the common region of all UGT1As was used to detect UGT1A expression. Total RNA from human liver was used as a control with UGT2B7 primers. Arrows indicate DNA bands of expected size whose sequence was confirmed. (B) Control RT-PCR analysis of total RNA from liver using indicated primers sets. (C) RT-PCR analysis of total RNA from the human melanoma cell line WM115 using indicated primers sets. Arrows indicate bands that were excised and sequenced. Band in UGT2B4 lane was found to be UGT2B7 by sequencing while UGT2B10 and UGT2B15 bands were confirmed to be expected UGTs. (D) RT-PCR analysis of total RNA from the human melanoma cell line WM3211. GAPDH was used as a positive control here to ensure cDNA quality. (E) Real-time PCR using TaqMan assays against indicated UGTs. ND = not detected.

While UGT expression has previously been reported in the skin [25], UGT expression in melanocytes had not been specifically addressed. In the present study, human melanocytes isolated from de-identified neonatal foreskins were analyzed for UGT expression by RT-PCR. Primer sets designed for individual UGT2B family members were used as well as one common primer set to detect any UGT1A family member. The UGT2As were not measured as they are largely found in the olfactory system [26]. As shown in Figure 1A, UGT2B7, UGT2B10 and UGT2B15 were found to be expressed in human melanocytes. The PCR products indicated by the black arrows (Figure 1A) ran at the predicted sizes, were excised and individual UGT sequences were confirmed. The band in the UGT2B4 lane (Figure 1A, Lane 1) was also excised and determined to be UGT2B7 by sequencing. This is not too surprising as the UGT2B family members share high homology. In fact, UGT2B4 and UGT2B7 share greater than 90% identity at the DNA level and strategies for specific primers is difficult. As a control, RT-PCR was performed using liver RNA to demonstrate that the UGT primers sets were functional and show predicted sizes for each UGT amplicon (Figure 1B). Expression of these same three UGT family members was also observed in human melanocytes from a second de-identified neonatal foreskin (Table 1; NOTE that the numbers 322 and 423 simply denote dates foreskin was obtained from Caucasian infants). Next, UGT expression was examined in several primary and metastatic melanoma cell lines by RT-PCR. Consistent with the UGT expression pattern observed in melanocytes, the primary melanoma cell line WM115 exhibited only UGT2B7, UGT2B10 and UGT2B15 expression (Figure 1C). Due to the high homology of family members, and similar to Figure 1A, the ‘UGT2B4’ band in Figure 1C was determined to be UGT2B7 after DNA sequencing. Also, the upper band in the UGT1A lane was excised and sequenced (although it was smaller than expected size) and was determined not to be any UGT family member. No UGT expression was observed in another primary melanoma cell line, WM3211 (Figure 1D), or in any of the three metastatic melanoma cell lines examined (Summarized in Table 1; Depicted in Figure S1). These results suggested that UGT expression is lost during melanoma progression.

**Table 1:** UGT mRNA expression in melanocytes and melanoma.

UGT	Melanocytes		PrimaryMelanoma		MetastaticMelanoma		
	322	423	WM115	WM3211	Lu1205	SKmel28	A375
UGT2B4	ND	ND	ND	ND	ND	ND	ND
**UGT2B7**	**+**	**+**	**+**	ND	ND	ND	ND
**UGT2B10**	**+**	**+**	**+**	ND	ND	ND	ND
UGT2B11	ND	ND	ND	ND	ND	ND	ND
**UGT2B15**	**+**	**+**	**+**	ND	ND	ND	ND
UGT2B17	ND	ND	ND	ND	ND	ND	ND
UGT2B28	ND	ND	ND	ND	ND	ND	ND
UGT1A	ND	ND	ND	ND	ND	ND	ND

ND = Not Detected.

In order to improve sensitivity and specificity of UGT mRNA detection, TaqMan gene expression assays were used for the rest of this report. These assays have been optimized to be specific for individual UGTs using a probe as well as a primer set. To illustrate this point, and re-affirm that UGT2B7 is present in melanocytes as opposed to UGT2B4, real-time PCR utilizing TagMan assays was performed on melanocyte cDNA. Figure 1E clearly shows that UGT2B7 is indeed expressed in human melanocytes and UGT2B4 was not detected.

### Re-expression of UGT2B7, UGT2B10 and UGT2B15 in Melanoma in Response to Anti-cancer Drugs

Considering that the UGTs are phase II metabolism enzymes and one of their major functions systematically is to eliminate drugs, we examined if the UGTs might play a role in inherent resistance of melanoma cells to chemotherapeutic agents. Thus, the melanoma cell line WM3211 was treated with various anti-cancer agents and UGT expression was assayed by real-time PCR. WM3211 was chosen for these experiments since it has no detectable UGT expression as determined by RT-PCR (Figure 1D).

**Figure 2:**
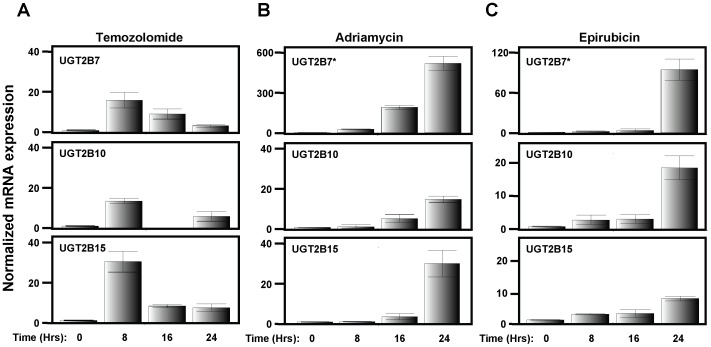
Re-expression of UGT2B7, UGT2B10 and UGT2B15 in WM3211 cells in response to anti-cancer agents. Predesigned Taqman gene expression assays were used to visualize individual UGT expression by real-time PCR following treatment of WM3211 cells with (A) 100 µM temozolomide, (B) 1.0 µM adriamycin or (C) 0.1 µM epirubicin. In all cases, UGT2B7, UGT2B10 and UGT2B15 expression was examined for the indicated anti-cancer agent at 0, 8, 16 and 24 hrs post treatment. * indicates different scale for y-axis.

First we treated WM3211 cells with temozolomide, which is currently indicated for treatment of metastatic melanoma. A dose of 100 µM was chosen for this experiment since published reports for temozolomide treatment of cell culture commonly use a minimum of 100 µM [27], [28]. As shown in Figure 2a, UGT2B7, UGT2B10 and UGT2B15 were re-expressed in WM3211 cells in response to temozolomide. Interestingly, no other UGTs were induced (data not shown), only the three UGT family members that are normally expressed in melanocytes (Table 1). The induction of UGT expression in response to temozolomide behaved in a time-dependent manner with maximal expression peaking at 8 Hrs after treatment (Figure 2A). For all three UGTs their expression declines after 8 Hrs, but their expression is still elevated 24 Hrs after treatment (Figure 2A).

To elucidate if the re-expression of these three UGT family members was specific for temozolomide or if this observed response may be a general mechanism for melanoma to defend itself against chemotherapeutics, the anti-cancer agents adriamycin and epirubicin were also examined. These two compounds were selected since they are closely related anthracyclines that are commonly used to treat various types of solid tumors although melanoma has proven to be resistant [29], [30]. Furthermore, epirubicin is known to be metabolized primarily by UGT2B7 [31] while the metabolism of adriamycin is much more complex and involves several different Phase II enzymes (including UGTs) and ABC transporters [30]. WM3211 melanoma cells were either untreated or treated with 0.1, 1.0 or 10 µM adriamycin (Figure S2A) or epirubicin (Figure S2B) and UGT expression was examined by real-time PCR after 8 Hrs. In both cases UGT2B7, UGT2B10 and UGT2B15 were observed to be re-expressed. Similar to temozolomide, no other UGT family member was induced (data not shown). Time courses examining UGT expression following treatment of WM3211 cells with 1.0 µM adriamycin (Figure 2B) or 0.1 µM epirubicin (Figure 2C) were then carried out. In both cases UGT2B7, UGT2B10 and UGT2B15 are induced in a time dependent manner, with the highest expression observed at 24 Hrs.

### Re-expression of UGT2B7, UGT2B10 and UGT2B15 in Metastatic Melanoma Cells in Response to Epirubicin and Vemurafenib

**Figure 3:**
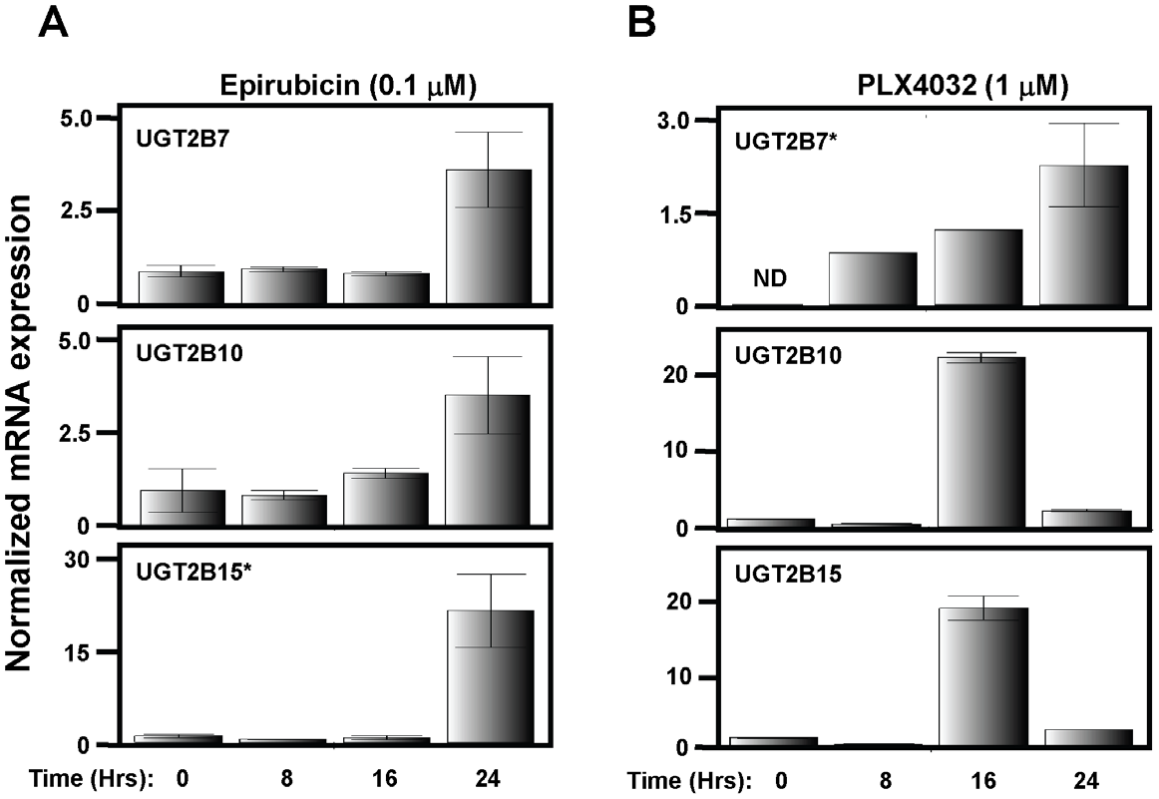
Re-expression of UGTs in SKmel28 cells in response to epirubicin and vemurafenib. Predesigned Taqman gene expression assays were used to visualize individual UGT expression by real-time PCR following treatment of SKmel28 cells with (A) epirubicin or (B) vemurafenib. Time course of UGT2B expression following epirubicin (100 nM) or vemurafenib (1 µM) treatment was examined at 0, 8, 16 and 24 hrs. * indicates different scale for y-axis. ND = not detected.

To ensure that the observed induction of UGTs was not limited to WM3211 cells, two metastatic melanoma cell lines (SKmel28 and A375) were examined for induction of UGT2B7, UGT2B10 and UGT2B15 in response to epirubicin. Time courses examining UGT expression following treatment of SKmel28 (Figure 3A) or A375 (Figure S3) with 0.1 µM epirubicin were then carried out. Once again, induction of all 3 UGTs was observed in both of these metastatic melanoma cell lines with maximal expression at 24 Hrs. Furthermore, since resistance has been observed in clinical trials with the promising new drug vemurafenib, we examined if the UGTs could be induced following vemurafenib treatment. SKmel28 cells, which harbor the BRAF V600E mutation, were treated with 1 µM vemurafenib, collected at 0, 8, 16 and 24 Hrs post treatment and assayed for UGT expression levels. As shown in Figure 3B, UGT2B7, UGT2B10 and UGT2B15 were all induced in response to vemurafenib.

### Increased Glucuronidation in Melanoma Cells Following Treatment with an Anti-cancer Agent

After demonstrating that UGTs can be re-expressed in melanoma cells following treatment with anti-cancer agents, the obvious question was whether UGT activity was restored as well. Therefore, glucuronidation was examined using the UGT-Glo Assay in melanoma cell lines that lack UGT expression and compared to the same cell lines after treatment with epirubicin. This assay employs a UGT Multienzyme Substrate which reacts with the luciferin detection reagent to give light that can be quantitated on a luminometer. However, if the substrate is glucuronidated then it will no longer react with the luciferin detection reagent to give light. Thus, two reactions are set up per sample, one with the co-substrate UDPGA and the other without. Only the reaction with the UDPGA will produce glucuronidated substrate if UGTs are present and active. In this manner the total UGT activity for the sample can be quantified by the difference in emitted light between the two reactions.

**Figure 4:**
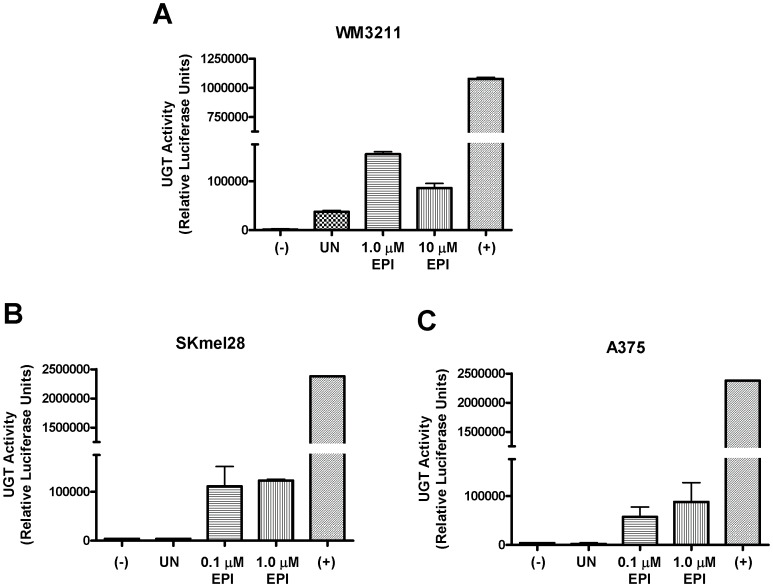
Induction of UGT activity by epirubicin. Glucuronidation activity was examined by the UGT-Glo Assay using 50 µg of homogenate per reaction in three melanoma cell lines; (A) WM3211 (B) SKmel28 and (C) A375 without treatment and compared to treatment with epirubicin (EPI) at the indicated concentrations for 24 hrs. (−) indicates a negative control in which no homogenate was present. (+) indicates use of human liver microsomes (known to have high concentrations of almost all UGT family members) as a positive control.

First, the primary melanoma WM3211 cells were examined for UGT activity. Cells were either left untreated (UN) or treated with epirubicin at indicated concentrations. Cells were harvested 24 hrs later and assayed for UGT activity. As shown in Figure 4A, UGT activity was increased in response to epirubicin treatment as compared to untreated cells. Interestingly, there was some basal glucuronidation activity in the untreated WM3211 cells indicating that UGTs are expressed in this cell line, but at levels below the detection of our RT-PCR experiment (Figure 1D). Human liver microsomes were used as a positive control as they have very high concentrations of UGTs and there was no homogenate present in the negative control to account for any background signal. Similar results were observed when this experiment was repeated for two metastatic melanoma cells lines, SKmel28 (Figure 4B) and A375 (Figure 4C). In both of these cell lines UGT activity was dramatically increased after treatment with epirubicin. In contrast to WM3211 cells, neither metastatic cell line exhibited UGT activity in the absence of treatment. Once again human liver microsomes were used as a positive control and the negative control lacked cell homogenate.

### UGT2B7 Knockdown Sensitizes WM115 Melanoma Cells to Anti-cancer Drugs

**Figure 5:**
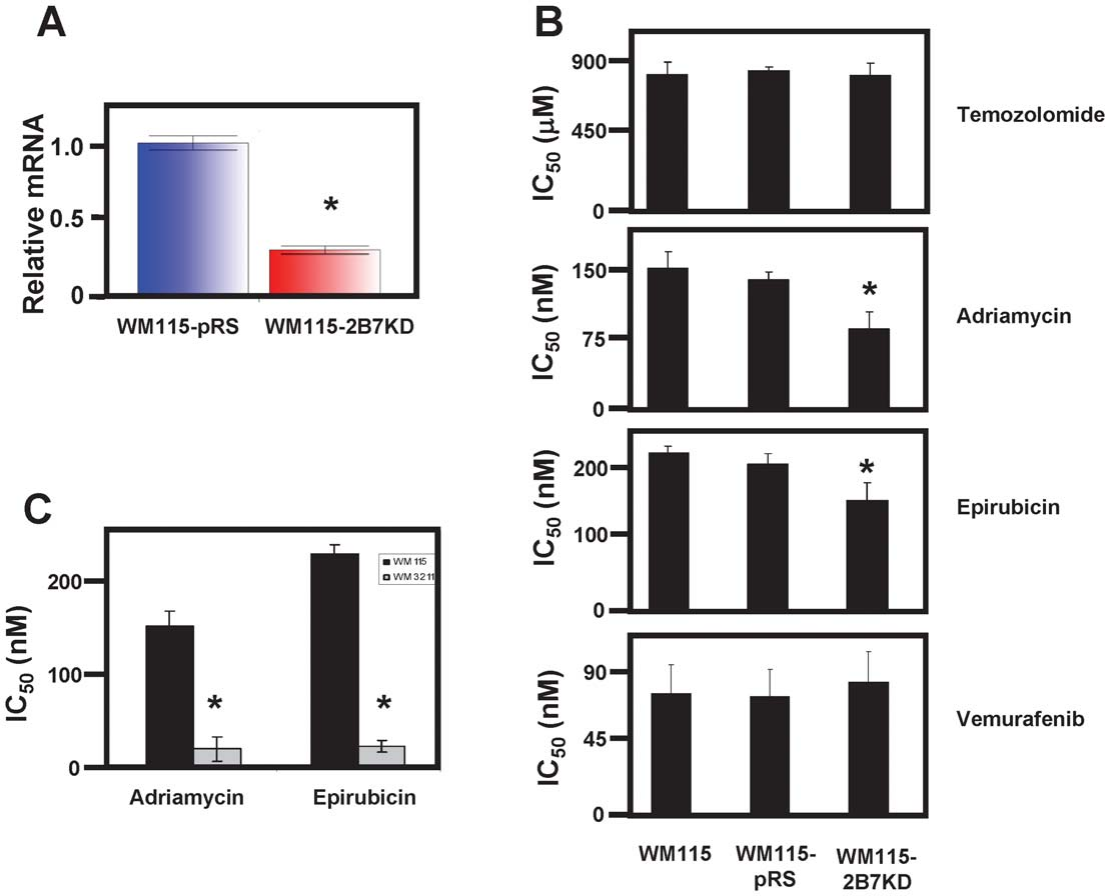
Knockdown of UGT2B7 sensitizes melanoma cells to adriamycin and epirubicin treatment. (A) Real-time PCR of UGT2B7 mRNA levels comparing melanoma cell lines stably expressing shRNA against UGT2B7 (WM115-2B7KD) or empty vector (WM115-pRS). (B) IC_50_ values determined from MTT assays assessing the contribution of UGT2B7 to melanoma resistance following treatment of temozolomide, adriamycin, epirubicin or vemurafeninb. (C) IC_50_ values determined from MTT assays assessing the sensitivity of melanoma cell lines with (WM115) and without (WM3211) UGT expression to anti-cancer drugs known to be metabolized by UGTs. All data graphs are the average of at least three independent experiments analyzed by GraphPad Prism. Error bars represent standard deviation and * indicates significance p<0.01 as compared to either control.

To ascertain the functional contribution of glucuronidation to melanoma resistance, UGT2B7 was knocked down in WM115 cells. shRNA directed against UGT2B7 was stably transfected into WM115 cells. This cell line was named WM115-2B7KD. Real-time PCR was then performed to confirm that UGT2B7 mRNA levels had been reduced. Figure 5A clearly shows that UGT2B7 mRNA levels in the WM115-2B7KD cell line was ∼60% less than that of WM115 cells stably transfected with empty vector (WM115-pRS). Next, half maximal inhibition concentrations (IC_50_) as determined by MTT assays, were performed to examine if knockdown of UGT2B7 sensitized WM115 cells to anti-cancer treatment. IC_50_ values for WM115-2B7KD were determined and compared to WM115-pRS (stable cell line made with empty vector as control) and the parental cell line against temozolomide, adriamycin and epirubicin. Consistent with a role for glucuronidation in melanoma resistance, WM115-2B7KD was found to have a significantly lower (p<0.01) IC_50_ value for adriamycin and epirubicin treatment as compared to both WM115 and WM115-pRS (Figure 5B) indicating that, indeed, knockdown of UGT2B7 sensitizes melanoma cells to these anti-cancer drugs. No effect on IC_50_ values was observed for temozolomide or vemurafenib treatment on the WM115-2B7KD cell line compared to the two control cell lines (Figure 5B).

If knocking down one UGT by ∼60% sensitized melanoma cells to adriamycin and epirubicin then it stands to reason that the IC_50_ for WM3211 melanoma cells (which lack UGT expression) would be significantly lower than WM115 parental cells (which have UGT expression). Thus, we compared the IC_50_s for WM115 and WM3211 directly for these drugs. As predicted, WM3211 cells were 7-fold and 9-fold more sensitive to adriamycin and epirubicin, respectively (Figure 5C).

## Discussion

The role of UGTs in melanoma etiology had not been investigated previously despite the UGTs being a major clearance mechanism for anti-cancer agents. In the present study UGT2B7, UGT2B10 and UGT2B15 were identified as being normally expressed in human melanocytes. The same three UGTs were found to be expressed in the primary melanoma cell line WM115. However, no UGT expression was detected in another primary melanoma cell line, WM3211, or in any of the three metastatic melanoma cell lines examined indicating that UGT expression is lost during melanoma progression. Interestingly we demonstrate that UGT2B7, UGT2B10 and UGT2B15 can be re-expressed in melanoma cells in response to the anti-cancer agents. The corresponding increase in UGT activity was also demonstrated following treatment. Thus, re-expression of the UGTs would presumably protect the cancer cells against anti-cancer drugs through enhanced metabolism and subsequent clearance. Importantly, these observations were consistent in both B-Raf wild type melanoma cells (WM3211) and B-Raf mutant cell lines (A375 and SKmel28).

Unfortunately, no commercially available antibody has been shown to detect endogenous UGT2B protein and, accordingly, our attempts to visualize endogenous UGT2B protein were unsuccessful. This is most likely due to the fact that UGTs are membrane bound, insoluble proteins. Accordingly, no full length human UGT has been purified and/or crystallized. In fact, the only crystal structure reported for a human UGT is the C-terminal half of UGT2B7 since the N-terminal half had to be cut off to solubilize the protein [32]. The vast majority of published UGT westerns are cell lines (usually insect) that are overexpressing recombinant UGTs. Further, a recent report clearly demonstrates that a large percentage of these overexpressed, recombinant UGTs are inactive [33]. One can easily imagine that the recombinant UGT proteins that are visible on westerns are not fully mature, active enzymes. Conversely, fully mature, active UGTs may not be visible on westerns even when overexpressed. Thus, consistent with several prior publications in the UGT field, the present study focused on mRNA expression of the UGT2B family members coupled with analysis of UGT activity. We utilized RNA interference and glucuronidation assays to examine the role of UGT2B7 on melanoma cell survival [1], [3], [24], [34], [35], [36], [37].

To test the role of glucuronidation in melanoma drug resistance directly, UGT2B7 was knocked down in WM115 cells, the only melanoma cell line we have identified with UGT expression thus far. Knockdown of UGT2B7 sensitized melanoma cells to epirubicin and adriamycin treatment, but had no effect on temozolomide or vemurafenib treatment. These results provide proof of principle that glucuronidation is involved in melanoma drug resistance *in vitro*. The most logical assumption is that UGT2B7 glucuronidates epirubicin and adriamycin metabolites directly resulting in its clearance before these drugs can exert their toxicity. This is consistent with prior reports that epirubicin is primarily metabolized by UGT2B7 [31] and that glucuronidation is also involved in adriamycin clearance [30]. The observation that the toxicity of temozolomide and vemurafenib was unchanged after UGT2B7 knockdown was not completely unexpected since UGTs have never been implicated in the metabolism of these drugs.

It is important to note that these experiments were done using a UGT2B7 knock down cell line, thus there was still some UGT2B7 present. Furthermore, UGT2B10 and UGT2B15 are still present. The effect of complete loss of UGT2B7 activity (or all UGT activity) would be predicted to have an even greater impact on the sensitization of melanoma to these anthracyclines. Moreover, the individual contributions of each UGT in melanoma survival warrants further investigation as do the underlying mechanism(s) controlling UGT silencing and re-expression in melanoma.

Although this is the first demonstration of UGT2B family members being induced in response to anti-cancer agents in cancer cells, this result is consistent with another report in which UGT1A1 and UGT1A10 expression levels were induced in response to the anti-cancer agent irinotecan in a lung cancer cell line [38]. The authors of that manuscript also demonstrated that the increased levels of UGT1A1 and UGT1A10 increased resistance of lung cancer cells to irinotecan [38]. More recently, another study demonstrated induction of UGT1A1 and UGT1A7 by irinotecan in colon cancer cells [39]. Thus, further investigation is warranted into the role of the UGTs as a general mechanism for intratumoral defense in human cancers.

With the novel demonstration that glucuronidation is involved in melanoma etiology and that active UGTs can be re-expressed in response to anti-cancer agents in a dose and time dependent manner, any future melanoma treatment strategy must account for intratumoral glucuronidation. This is of particular importance if the drug is a known UGT substrate. Inclusion of a UGT inhibitor as part of a cocktail to eliminate the contribution of glucuronidation to intratumoral resistance may be a viable strategy. In support of this idea, one previous study demonstrated that valproic acid can sensitize A375 metastatic melanoma cells to epirubicin [40]. What the authors did not know (or at least did not convey) is that valproic acid is used as a competitive inhibitor for UGT2B7 [41]. Thus, addition of valproic acid most likely sensitized A375 cells to epirubicin by competitively inhibiting the glucuronidation activity of the re-expressed UGT2B7.

Great strides have been made recently regarding our understanding of melanoma resistance thanks to recent intense efforts to understand the acquired resistance to vemurafenib observed in the clinic. These collective efforts have yielded a wealth of information (reviewed nicely in [42]). Several independent labs have demonstrated that melanoma can acquire resistance to vemurafenib in various ways, including mutation of NRAS, upregulation of CRAF or ARAF, redundant PI3-Kinase signaling or activation of COT [42]. Clearly, melanoma has a multitude of acquired resistance schemes it can employ and we humbly submit that induction of the UGT2Bs is another piece to an extraordinary puzzle. Further, now that this vemurafenib has been approved for melanoma treatment in the United States and Europe, the race is on to find the most effective combinatorial treatment to overcome this acquired resistance and combat this deadly disease. To be clear, we do not advocate that re-expression of the UGTs in melanoma in response to vemurafenib constitutes a mechanism of resistance to this drug *per se*, but we do argue that the ideal candidate to use in combination with vemurafenib should not be a UGT2B substrate.

In summary, we have identified three UGTs as being normally expressed in melanocytes and their expression appears to be lost during melanoma progression. In response to anti-cancer drugs, these same three UGTs are re-expressed in melanoma cells. The corresponding increase in glucuronidation was also observed and, thus, we propose that this re-expression constitutes an intratumoral defense mechanism involved in melanoma resistance to anti-cancer drugs. This hypothesis is strengthened by demonstration that knockdown of UGT2B7 in melanoma cells sensitized these cells to anti-cancer agents known to be metabolized by glucuronidation. Taken together, these novel observations ensconce UGTs in melanoma etiology and entreat that UGTs must be taken into account for all future melanoma treatment strategies.

## Supporting Information

Figure S1 **Absence of UGT mRNA expression in metastatic melanoma.** RT-PCR analysis of cDNA reverse transcribed from total RNA from the human metastatic melanoma cell lines A375 (A), Lu1205 (B) and SKmel28 (C) using indicated primers sets. GAPDH primers were used as a positive control.
(PDF)

Figure S2 **Dose-dependent re-expression of UGT2B7, UGT2B10 and UGT2B15 in WM3211 cells in response to anti-cancer agents.** Predesigned Taqman gene expression assays were used to visualize individual UGTexpression by real-time PCR following treatment of WM3211 cells with (A) adriamycin or (B) epirubicin. In both cases, UGT2B7, UGT2B10 and UGT2B15 expression was examined following treatment of 0, 0.1, 1.0 or 10 µM of indicated drug 8 hrs post treatment. *indicates different scale for y-axis.
(PDF)

Figure S3 **Re-expression of UGT2B7, UGT2B10 and UGT2B15 in A375 cells in response to epirubicin.** Predesigned Taqman gene expression assays were used to visualize individual UGT expression by real-time PCR following treatment of A375 cells with epirubicin. Time course of indicated UGT2B expression following epirubicin treatment (100 nM) was examined at 0, 8, 16 and 24 hrs. * indicates different scale for y-axis.
(PDF)

Table S1 Primers used in conventional PCR experiments. Both sense and anti-sense primers used to distinguish individual UGT family members are listed along with the size of the amplicon. These primers were used in Figure 1A–D and Figure S1.
(PDF)
