# Supplementary figures and images for: Anti-Cancer Drugs Elicit Re-Expression of UDP-Glucuronosyltransferases in Melanoma Cells
Source: PLoS One. 2012 Oct 22;7(10):e47696. doi: 10.1371/journal.pone.0047696 (PMC3478267; doi:10.1371/journal.pone.0047696)

**Figure S1**

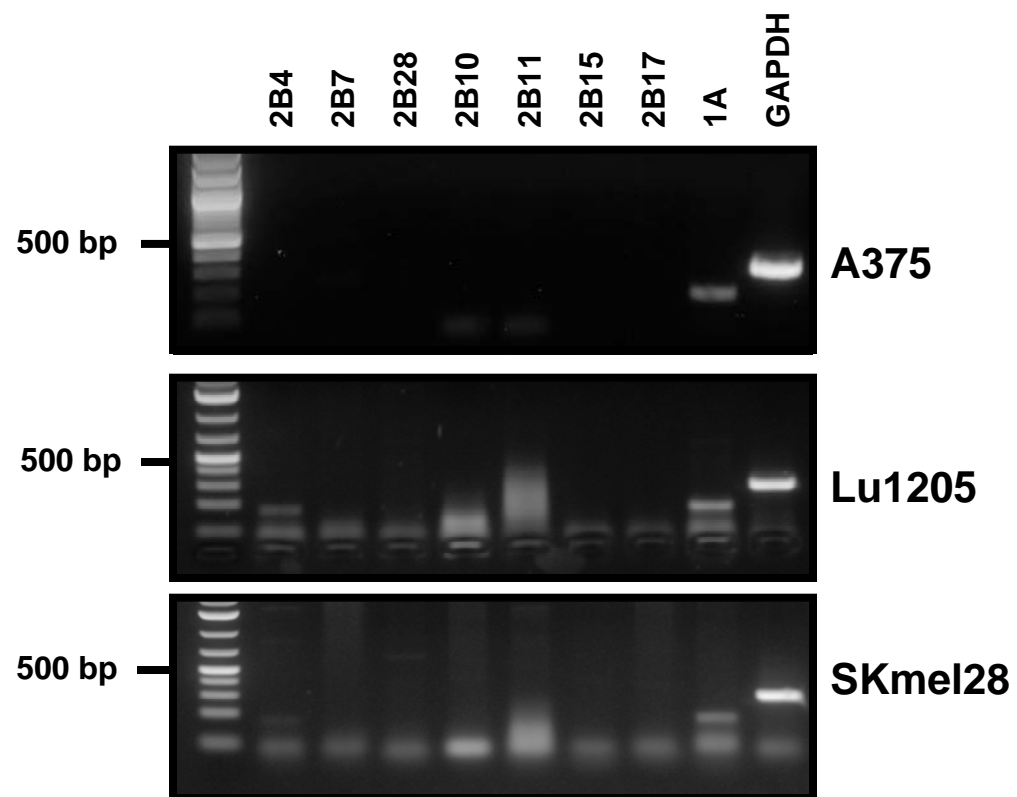

Supplement: Figure S1 — Absence of UGT mRNA expression in metastatic melanoma. RT-PCR analysis of cDNA reverse transcribed from total RNA from the human metastatic melanoma cell lines A375 (A), Lu1205 (B) and SKmel28 (C) using indicated primers sets. GAPDH primers were used as a positive control. (PDF) [file pone.0047696.s001.pdf]

**Figure S2**

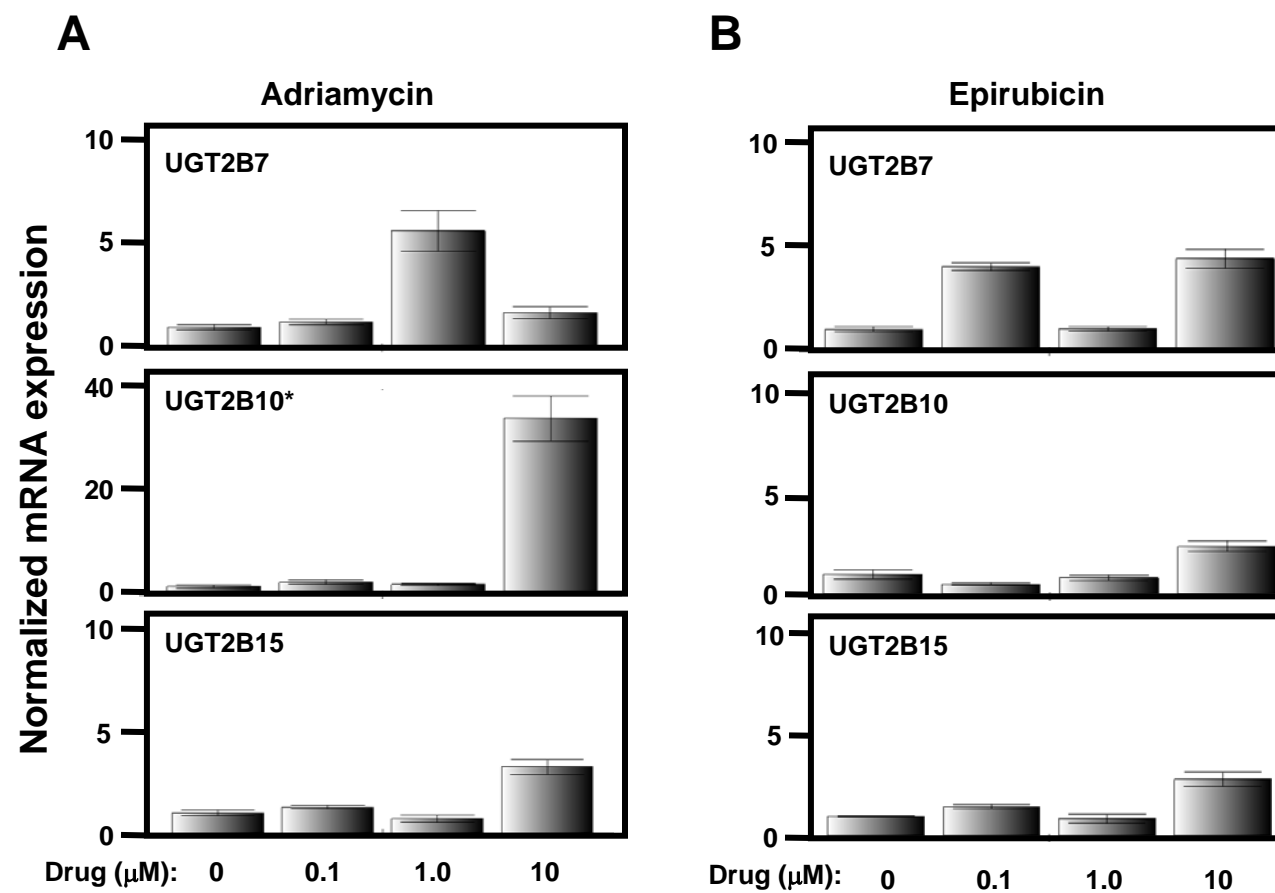

Supplement: Figure S2 — Dose-dependent re-expression of UGT2B7, UGT2B10 and UGT2B15 in WM3211 cells in response to anti-cancer agents. Predesigned Taqman gene expression assays were used to visualize individual UGTexpression by real-time PCR following treatment of WM3211 cells with (A) adriamycin or (B) epirubicin. In both cases, UGT2B7, UGT2B10 and UGT2B15 expression was examined following treatment of 0, 0.1, 1.0 or 10 µM of indicated drug 8 hrs post treatment. *indicates different scale for y-axis. (PDF) [file pone.0047696.s002.pdf]

**Figure S3**

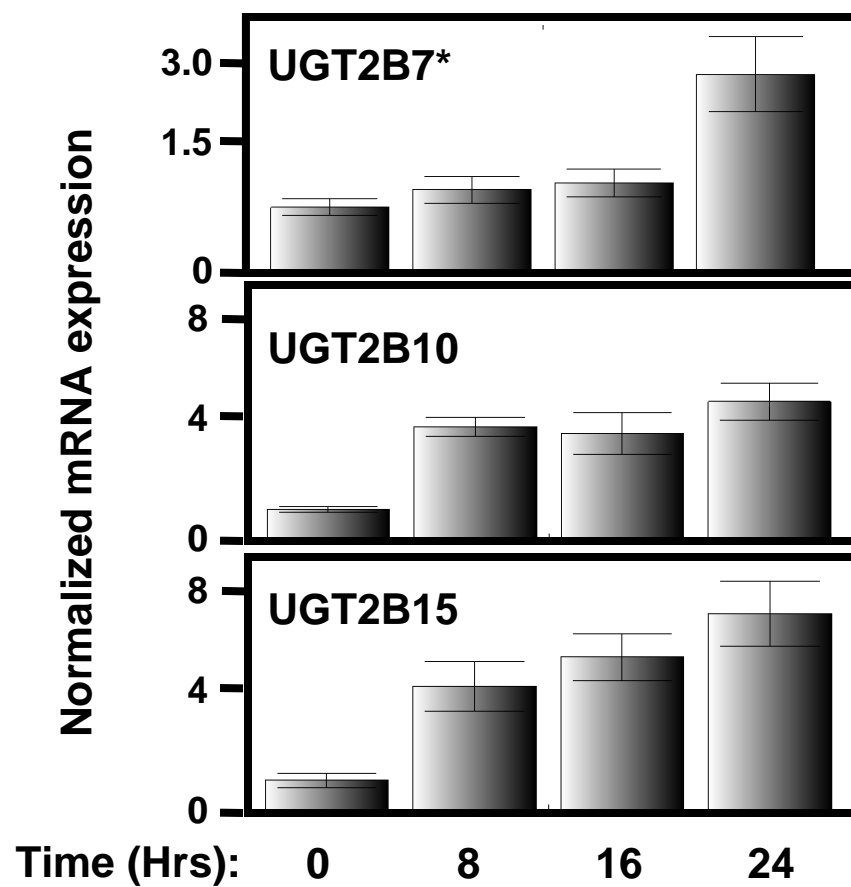

Supplement: Figure S3 — Re-expression of UGT2B7, UGT2B10 and UGT2B15 in A375 cells in response to epirubicin. Predesigned Taqman gene expression assays were used to visualize individual UGT expression by real-time PCR following treatment of A375 cells with epirubicin. Time course of indicated UGT2B expression following epirubicin treatment (100 nM) was examined at 0, 8, 16 and 24 hrs. * indicates different scale for y-axis. (PDF) [file pone.0047696.s003.pdf]
