# Supplementary material for: Anti-Cancer Drugs Elicit Re-Expression of UDP-Glucuronosyltransferases in Melanoma Cells
Source: PLoS One. 2012 Oct 22;7(10):e47696. doi: 10.1371/journal.pone.0047696 (PMC3478267; doi:10.1371/journal.pone.0047696)
Supplement: Table S1 — Primers used in conventional PCR experiments. Both sense and anti-sense primers used to distinguish individual UGT family members are listed along with the size of the amplicon. These primers were used in Figure 1A–D and Figure S1. (PDF) [file pone.0047696.s004.pdf]

Table S1

Supplemental Table S1. Primers used in conventional PCR experiments

GENE	Sense Primer	Anti-Sense Primer	Expected Size (bps)
UGT2B4	**5'-**CTCTCCTGGCTACGCAA-**3'**	**5'-**TTTGCAGTGGAGTCCTCC-**3'**	326
UGT2B7	**5'-**CTTCTCTCCTGGCTACACTT-**3'**	**5'-**TTTGCAGTGGAGTCCTCC-**3'**	329
UGT2B10	**5'-**CTGGCTGAGCTATTTAACATACCC-**3'**	**5'-**CGCATAAGCCATATGTCAGC-**3'**	298
UGT2B11	**5'-**GACATCGTTTTTGCAGATGCTGT-**3'**	**5'-**CGCATAAGCCATATGTCAGC-**3'**	306
UGT2B15	**5'-**GTTGGGAATATTATGACTACAGTAAC-**3'**	**5'-**TTTGGTAAGAATGGGCGAG-**3'**	462
UGT2B17	**5'-**GTGTTGGGAATATTCTGACTATAATATA-**3'**	**5'-**TTTGGTAAGAATGGGCGAG-**3'**	462
UGT2B28	**5'-**CAGTCTCTGCTTCACTCC-**3'**	**5'-**TTTGCAGTGGAGTCCTCC-**3'**	322
UGT1A	**5'-**TCCTGTGGCGGTACACTG-**3'**	**5'-**TCAATGGGTCTTGGATTTGTGG-**3'**	600
GAPDH	**5'-**CCAGGGCTGCTTTTAACTC-**3'**	**5'-**GCTCCCCCCTGCAAATGA-**3'**	291
